# Visual acuity improvements after implantation of toric intraocular lenses in cataract patients with astigmatism: a systematic review

**DOI:** 10.1186/1471-2415-12-41

**Published:** 2012-08-15

**Authors:** Blaise Agresta, Michael C Knorz, Christina Donatti, Daniel Jackson

**Affiliations:** 1 Health Economics and Outcomes Research, IMS Health, London, UK; 2 FreeVis LASIK Center, Medical University of Mannheim, Mannheim, Germany; 3 Market Access and Reimbursement, Alcon Laboratories, Inc, Cointrin, Switzerland

**Keywords:** Astigmatism, Toric, Intraocular lenses, Uncorrected distance visual acuity, Uncorrected near visual acuity, Visual acuity, Systematic review, Cataracts

## Abstract

**Background:**

Cataracts are a common and significant cause of visual impairment globally. We aimed to evaluate uncorrected distance visual acuity (UDVA) as an outcome in treating astigmatic cataract patients to assist clinicians or ophthalmologists in their decision making process regarding available interventions.

**Methods:**

Medline, Embase and Evidence Based Reviews were systematically reviewed to identify relevant studies reporting changes in UDVA, UIVA and UNVA after cataract surgery in presbyopic patients. Strict inclusion/exclusion criteria were used to exclude any non-relevant studies. Relevant outcomes (UDVA, UIVA and UNVA) were identified from the studies retrieved through the systematic review process.

**Results:**

The systematic review identified 11 studies which reported UCVA. All 11 studies reported UDVA. Four brands of toric intraocular lenses (IOLs) were reported in these studies. All studies identified in the literature search reported improvements in UDVA following surgical implant of a toric IOL. The largest improvements in VA were reported using the Human Optics MicroSil toric IOL (0.74 LogMAR, UDVA) and the smallest improvements were also reported using the Human Optics MicroSil toric IOL (0.23 LogMAR, UDVA) in a different study.

**Conclusions:**

The results of this systematic review showed the aggregate of studies reporting a beneficial increase in UDVA with the use of toric IOLs in cataract patients with astigmatism.

## Background

The main cause of the formation of cataracts is age, but can also be ocular and systemic diseases (diabetes and uveitis), systemic medications (steroids and phenothiazines), trauma, ionizing radiation (X-ray and UV light), congenital diseases and inherited abnormalities (myotonic dystrophy, Marfan’s syndrome, Lowe’s syndrome, rubella) [1].

Cataracts are a common and significant cause of visual impairment globally. Cataract surgery is one of the most commonly performed surgeries in the United Kingdom (UK) National Health Service (NHS). In the UK, 10% of persons aged 65 years and over have received cataract surgery and 30% of this population (≥ 65 years) have been found to have a visually impairing cataract in one or both eyes [2]. The prevalence of cataract eye surgery increases with age, from 16% in the 65 to 69 age group to 71% in those aged 85 years or more and tends to affect women more than men. In the UK it is estimated that 2.4 million people aged 65 or older have a visually impairing cataract in one or both eyes and a further 225,000 new cases are predicted annually [3].

Astigmatism occurs when the patient’s cornea is steeper in the vertical axis (with-the-rule astigmatism) or in the horizontal axis (against-the-rule astigmatism) [4] when the principal meridians are perpendicular. A third type of regular astigmatism, oblique astigmatism, occurs when the steepest curve lies between 120–150° and 30–60°. When replacing a lens during cataract surgery, astigmatism can either be corrected by prescription glasses, contact lenses, corneal relaxing incisions, astigmatic keratotomies, limbal relaxing incisions, excimer laser ablation, and toric IOL implantation.

Astigmatism and cataracts reduce the quality of life of a patient [5,6]. The use of a toric intraocular lens (toric IOL) is designed to replace the cataractous lens of an eye and to correct the corneal astigmatism. Thus if toric IOLs are safe and efficient, an increase in quality of life should be observed in cataract patients with astigmatism. This increase in quality of life would not have been as significant had only cataracts or astigmatism alone been treated in this patient population.

This systematic review attempts to identify and describe published literature reporting the postoperative UDVA, UIVA, and UNVA outcomes in patients undergoing cataract surgery with astigmatism and to show how close patients can achieve normal vision with the use of all toric IOLs.

## Results

### Results of the systematic review

**Figure 1:**
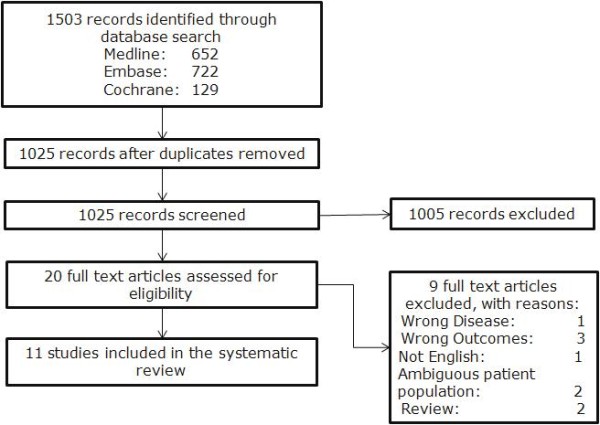
QUROUM chart describing inclusion and exclusion of studies included in this systematic review.

The systematic review identified 11 studies that measured uncorrected visual acuity (Figure 1). Initially, UNVA and UIVA were also included in the analysis, but no studies were identified that reported UNVA or UIVA. Of the 11 studies that reported UDVA, 2 randomised controlled trials were identified, 7 observational studies, 1 prospective cohort study, and 1 retrospective observational. Using the Oxford centre for evidence based medicine–grades of evidence, 9 studies were classified as Level IIIb, and two studies categorized as Ib. A range of different outcome measurement units were reported, including using the decimal of the Snellen measurement, and the logarithm of the minimal angle of resolution (LogMAR).

Four brands of MIOLs were identified in these studies. These brands were Human optic MicroSil (6116TU), Rayner T-Flex (623T), AcrySof Toric (SN60T3, SN60T4, SN60T5) and Staar Toric (AA4203T).

This analysis showed gains in improvement for UDVA (Table 1). Post-operative UDVA values were closer to a normal sight score (20/20 Snellen) in 4 models of toric IOLs tested across the 11 studies.

**Table 1:** Results of the systematic review

**Author**	**Study Type (Grade)**	**Lens**	**Unit Measurement**	**N (eyes)**	**Uncorrected Distance Visual Acuity**		
					**Preoperative**	**Postoperative**	**P**
De Silva (2006) [7]	Observational (IIb)	Human Optics MicroSil 6116TU	LogMAR–6 months	21	0.55 ± 0.34	0.32 ± 0.24	NR
Entabi (2011) [8]	Observational (IIb)	Rayner T-Flex 623T	LogMAR–4 months	33	–	0.28 ± 0.23	NR
Kersey (2007) [9]	Observational (IIb)	Human Optics MicroSil 6116TU	LogMAR–9-19 months	7	1.31 ± 0.65	0.39 ± 1.15	NR
Kim (2010) [10]	Observational (IIb)	AcrySof Toric	LogMAR–13.3 months (mean)	30	0.87 ± 0.67	0.33 ± 0.18	<0.05
Koshy (2010) [11]	Observational (IIb)	AcrySof Toric SN60TT	LogMAR–6 months	30	–	0.2	NR
Lane (2009) [12]	Prospective (IIb)	AcrySof Toric	LogMAR–6 months	40	–	−0.07	NR
Leyland (2001) [13]	Observational (IIb)	Staar Toric	LogMAR–8 weeks	22	1.45 ± 0.64	0.27 ± 0.25	NR
Mendicute (2009) [14]	RCT (Ib)	AcrySof Toric SN60T3, SN60T4, SN60T5	LogMAR–3 months	40	–	0.11 ± 0.15	NR
Mendicute (2008) [15]	Observational (IIb)	AcrySof Toric SN60T3, SN60T4, SN60T5	LogMAR–3 months	30	–	0.16 ± 0.18	NR
Mingo-Botin (2010) [16]	RCT (Ib)	AcrySof Toric	LogMAR–3 months	40	0.72 ± 0.26	0.13 ± 0.10	NR
Ruhswurm (2000) [17]	Retrospective observational (IIb)	Staar Toric AA4203T	Decimal–3-62.8 months	37	–	0.61 ± 0.29 (0.21LogMAR)	NR

NR, Not reported.

Outcomes associated with astigmatism are reported in Table 2. When reported within the 11 identified studies, changes in refractive sphere, spherical equivalent, refractive astigmatism and keratometric astigmatism along with the mean age of the patients and the surgical incision used to implant the toric IOLs are shown.

**Table 2:** Astigmatic outcomes reported in studies identified in the systematic review

**Author**	**Mean age**	**Incision**	**Refractive Sphere**		**Spherical Equivalent**		**Refractive Astigmatism**		**Keratometric Astigmatism**	
			**Pre operative**	**Post operative**	**Pre operative**	**Post operative**	**Pre operative**	**Post operative**	**Pre operative**	**Post operative**
De Silva (2006) [7]	76.1 ± 11.4	3.4 mm temporal corneal incision	2.58 ± 3.14	−0.13 ± 1.00	0.82 ± 3.28	−1.07 ± 0.82	3.52 ± 1.11	1.23 ± 0.90	3.08 ± 0.76	2.90 ± 1.52
Entabi (2011) [8]	80.6 ± 8.9	3.2 mm steep meridian corneal incision	1.55 ± 2.42	0.13 ± 0.72	−0.13 ± 2.31	−0.26 ± 0.62	3.35 ± 1.20	0.95 ± 0.66	2.94 ± 0.89	2.42 ± 1.38
Kersey (2007) [9]	62	2.7 mm clear corneal temporal incision	–	–	–	−0.36	7.36	–	10.12 ± 5.60	2.27 ± 1.14
Kim (2010) [10]	55.8 ± 16.2	2.2 mm temporal clear corneal incision	−1.88 ± 3.58	–	19.8 ± 2.10	–	1.28 ± 0.72	0.28 ± 0.21	Manual keratometer 1.57 ± 0.51 Optical coherence biometry (IOL master) 1.63 ± 0.43 Scheimpflug SimKs (Pentacam) 1.67 ± 0.60	–
Koshy (2010) [11]	71.5	3.0 mm temporal self-sealing incision	–	–	–	–	–	–	Partial coherence interferometry 1.97 ± 0.58 Scheimpflug SimKs (Pentacam) −1.84 ± 0.79	−0.84 ± 0.41
Lane (2009) [12]	69.1 ± 11.9	3.0 mm temporal incision	–	–	–	–	–	–	–	–
Leyland (2001) [13]	77	3.0 mm temporal incision	–	–	–	–	3.2 ± 1.1	0.85 ± 0.66	–	–
Mendicute (2009) [14]	69.3 ± 8.2	2.75 mm temporal corneal incision	0.88 ± 1.80	0.71 ± 1.85	–	–	−1.75 ± 0.71	−0.62 ± 0.46	–	–
Mendicute (2008) [15]	72.1 ± 8.2	2.75 mm temporal corneal incision	1.26 ± 1.85	−0.12 ± 0.44	–	–	−2.34 ± 1.28	−0.72 ± 0.43	–	–
Mingo-Botin (2010) [16]	71.5 ± 11.1	2.8 mm corneal incision (temporal for right eye; nasal for left eye)	−0.40 ± 1.89	0.20 ± 0.33	−0.21 ± 0.12	–	−1.89 ± 0.57	−0.61 ± 0.41	1.73 ± 0.38	1.87 ± 0.66
Ruhswurm (2000) [17]	75 ± 9	3.0–3.2 mm clear corneal self-sealing incision	–	–	–	–	2.68 ± 0.93	0.84 ± 0.63	2.7 ± 0.88	2.3 ± 0.80

The greatest changes in refractive sphere were reported by De Silva using the Human Optic MicroSil toric IOL with a change of 2.71. Reported pre/post-operative differences in spherical equivalent changes were also greatest in the De Silva study (an adjustment of 1.89). Entabi reported the greatest difference in refractive astigmatism (an adjustment of 2.4), using the Rayner T-Flex toric IOL, and Kersey reported the greatest change in keratometric astigmatism (an adjustment of 7.85) using the Human Optics MicroSil toric IOL.

The reported surgically induced astigmatism (SIA), rotational stability and if additional surgery was needed within the identified studies are shown in Table 3.

**Table 3:** Surgically inducted astigmatism and rotational stability outcomes of toric lenses identified in the systematic review

**Authors**	**Surgically inducted astigmatism**	**Rotational Stability**	**2**^**nd**^** surgery needed**
De Silva (2006) [7]	Not reported	5 degrees (range: 0–15)	1
Entabi (2011) [8]	−2.95 ± 1.34	3.4 degrees (range: 1–12)	1
Kersey (2007) [9]	Not reported	5.33 degrees (range: 0–9)	NR
Kim (2010) [10]	Not reported	3.45 degrees (range: 0–10.3)	0
Koshy (2010) [11]	Not reported	2.66 degrees(range: 0–7.6)	NR
Lane (2009) [12]	Not reported	NR	NR
Leyland (2001) [13]	0.79 (0.22)*	8.9 degrees (range: 0–40)	NR
Mendicute (2009) [14]	J0 = 100% within ±1.0D; J45 = 100% within ±0.5D	3.53 degrees (range:0–8)	0
Mendicute (2008) [15]	J0 = 100% within ±1.0D; J45 = 100% within ±1.0D	3.63 degrees (range: 0–12)	0
Mingo-Botin (2010) [16]	J0 = 100% within ±0.5D; J45 = 90% within ±0.5D; M = 90% within ±0.5D	3.65 (range: 0–10)	0
Ruhswurm (2000) [17]	78.4% within ±1.0D; 48.6% within ±0.5D	100% >25 degrees	NR

* magnitude of SIRC as % or IRC.

NR, Not Reported.

### Outcomes of the systematic review

The greatest increase in VA was reported by Kersey (2007), investigating the efficacy of the Human Optics MicroSil [9] with an increase of 0.92 LogMAR in UDVA. The smallest improvements in VA were reported by De Silva (2006). UDVA increased by only 0.23 LogMAR [7].

Of the patient study groups that were identified in the systematic search, the prospective study by Lane (2009) reported the mean VA with the closest value to normal VA (20/20) when measuring UDVA [12] with a postoperative UDVA of −0.07 LogMAR [12].

The trial by Kersey (2007), an observational study measuring the VA in the Human Optics MicroSil toric IOL measured the least gain in UDVA with a mean post-operative result of 0.39 LogMAR [9]. It should be noted that the population in this trial also had the largest variation compared with other identified studies probably due to the small patient population (N = 7). This population also had the largest mean degree of astigmatism (10.12°), which also allocates a smaller error accepted during the toric IOL positioning. This again was an outlier, with the highest reported mean corneal astigmatism after the Kersey trial, was reported by De Silva (2006), with a mean of 3.08.

## Methods

### Systematic search

The systematic review was conducted to evaluate the efficacy of toric IOLs in astigmatic cataract patients. Databases used in the search included: Medline, Medline In-Process (from 1948 to present), Embase (from 1988 to 2011) and Evidence Based Reviews were accessed via the OVID platform to search for relevant studies. The search terms used in search included: lens diseases, cataract, aphakia, cataract extraction, toric, lens implantation, lenses-intraocular, and astigmatism (see Additional file 1: Appendix A for full search results). An analysis of how the individual studies identified in the literature search were excluded was achieved using the QUORUM (Quality of Reporting of Meta-analyses) chart (Figure 1). Inclusion/exclusion criteria were used to systematically exclude any studies that were not relevant for the review. Accommodative lenses were not the focus of this review, and were excluded from the search. Studies that published results in a language other than English were also excluded. Data extraction was completed using a template created in Microsoft Excel. Data was extracted directly from the information reported in the published journal articles. Demographic data and study details (number of patients, study time length, patient population, interventions, comparators) and study outcomes were extracted. Baseline characteristics and the severity of the astigmatisms within the patient populations were also extracted. The Oxford Center for Evidence-based Medicine–Grades of Evidence [18] was used to grade the identified studies.

### Outcomes reviewed

This analysis focused on UDVA. UIVA, and UNVA were initially included in the analysis. The safety of toric IOLs and the change in quality of life due to toric IOLs were not analysed in this review.

### Review restrictions

The systematic review was restricted to a cataract population with astigmatism, treated with toric lens, and with the outcome, uncorrected visual acuity, reported.

Studies that did not specify a cataract population were assumed to be a mixed patient population (cataract patients and refractive lens exchange), and excluded from the review.

Either uncorrected or best corrected visual acuity can be used to measure improvements in VA. While the WHO may define visual impairment and blindness according to visual acuity with “best possible correction”, evidence suggests that uncorrected visual acuity has a significant impact on vision-related quality of life [19,20]. Before quality of life improvements caused by toric IOLs in astigmatic cataract patients are assessed, it is necessary to demonstrate the efficacy of toric IOLs.

The measurement unit of uncorrected visual acuity varies across many studies. The Snellen scale, Jaeger scale, the decimal of the Snellen scale and the logarithm of the minimum angle of resolution (LogMAR) are the most commonly used units of measurement [21]. Converting these units into a standardized unit has statistical challenges where individual (patient) level data is not available [21]. Published literature exists to suggest that transforming group-level mean and standard deviation of VA across different levels of measurement is possible [21]. But to transform group-level data, a ‘reasonable size’ patient population (N ≥ 30) is needed [21].

Studies that did not report the outcomes of the full population were not included, and excluded on the basis of incomplete data.

## Discussion

Our study compared uncorrected visual acuity, measured both at near, intermediate and distant ranges. The results of studies identified through this systematic review showed improvements in UDVA. Prior to this study, there have been no known evaluations comparing studies measuring outcomes in toric intraocular lens. This study shows the collective improvement of distant visual acuity.

Toric IOLs have a two-function purpose. The first is to restore visual acuity deteriorated by cataract of the eye. The second function of the toric IOL is to correct corneal astigmatism, thereby restoring vision to a level close to normal, where spectacles are no longer needed. Hence, the authors argue that uncorrected visual acuity, as an outcome measure, is more important than best corrected visual acuity when measuring the efficacy of toric IOLs. A final outcome to be included (not included in this analysis) would be whether patients become spectacle independent, and the impact this has on the quality of life of the patient population.

The outcomes of this systematic review showed significant improvements in uncorrected distant visual acuity. While only a half of the studies (5/11) reported pre- and post-operative values, the outcomes of trials only reporting post-operative values were similar to the post-operative results in the studies that reported pre- and post operative values. The trials that were identified in the systematic search, only one reported QoL, and spectacle independence [16]. Using the VF-14 instrument to measure QoL, patient satisfaction increased from 60.13 (±17.14) to 90.73 (±11.07). Spectacle independence decreased for patients treated with toric IOLs from 40% (who always used spectacles) to 0% (who always used spectacles). Patients who occasionally wore spectacles decreased from 20% to 10% and patients who frequently wore spectacles decreased from 30% to 5%. Patients that never wore spectacles increased from 15% to 85%.

## Conclusions

This systematic review has provided evidence to support the hypothesis that toric IOLs increase the uncorrected distant visual acuity in cataract patients. A major issue with the supporting evidence is the lack of consistency among studies reporting toric IOL outcomes. Uncorrected visual acuity is one of the many possible outcomes that can be used to measure the efficacy of toric IOLs, and there have been many studies reporting variations in methods of reporting such results. Mean visual acuity after cataract surgery is the most commonly reported method, while proportions of patients achieving a minimum visual acuity level have been another highly reported method. Unfortunately, few studies have been published that report UDVA using toric lens in a cataract population with astigmatism, and no studies were identified that reported either UIVA or UNVA. Challenges exist in comparing this proportional method of reporting with other studies.

## Abbreviations

IOL, Intraocular lenses; NHS, National Health Service; NR, Not reported; QoL, Quality of life; QUORUM, Quality of reporting of meta-analyses; UDVA, Uncorrected distance visual acuity; UIVA, Uncorrected intermediate visual acuity; UK, United Kingdom; UNVA, Uncorrected near visual acuity; VA, Visual acuity.

## Competing interests

Blaise Agresta and Christina Donatti were paid consultants to Alcon Laboratories, Inc. and employees of IMS Health. Daniel Jackson is an employee of Alcon Laboratories, Inc. Michael C. Knorz is a consultant to Alcon.

## Authors’ contributions

BA participated in the design and review of the systematic review, managed the data collection and was involved with the manuscript development. CD and DJ provided technical expertise for the analysis and interpretation of the data and were involved with the manuscript development. MK provided clinical expertise and approved the final version of the manuscript. All authors read and approved the final manuscript.

## Pre-publication history

The pre-publication history for this paper can be accessed here:

http://www.biomedcentral.com/1471-2415/12/41/prepub

## Supplementary Material

Additional file 1 Appendix A. Search string. Click here for file
