# Supplementary material for: Visual acuity improvements after implantation of toric intraocular lenses in cataract patients with astigmatism: a systematic review
Source: BMC Ophthalmol. 2012 Aug 15;12:41. doi: 10.1186/1471-2415-12-41 (PMC3480885; doi:10.1186/1471-2415-12-41)
Supplement: Additional file 1 — Appendix A. Search string. [file 1471-2415-12-41-S1.doc]

Appendix A: Search string

| ***#*** | ***Searches*** | ***Medline*** | ***Embase*** | ***Cochrane*** |
| --- | --- | --- | --- | --- |
| 1 | Lens Diseases/ | 631 | 906 | 10 |
| 2 | exp Lens diseases/ | 23276 | 38553 | 573 |
| 3 | Cataract/ | 20056 | 25001 | 524 |
| 4 | exp Aphakia/ | 2151 | 1967 | 44 |
| 5 | aphakia$.tw. | 1624 | 983 | 53 |
| 6 | cataract$.tw. | 35258 | 30565 | 3584 |
| 7 | (Lens adj3 diseas$).tw. | 118 | 108 | 24 |
| 8 | or/1-7 | 44184 | 50691 | 3739 |
| 9 | exp cataract extraction/ | 24145 | 21780 | 1922 |
| 10 | (cataract$ adj3 extract$).tw. | 6550 | 4869 | 937 |
| 11 | (cataract$ adj3 (extract$ or remov$ or surg$)).tw. | 16171 | 14855 | 2691 |
| 12 | or/9-11 | 27832 | 24446 | 3191 |
| 13 | (Multifocal$ or multi-focal$ or bifocal$ or Bi-focal$ or varifocal$ or vari-focal$).tw. | 18410 | 18962 | 576 |
| 14 | Lens Implantation, Intraocular/ | 6076 | 4169 | 713 |
| 15 | exp Lenses-Intraocular/ | 11148 | 12237 | 731 |
| 16 | (Intraocul$ adj3 lens$).tw. | 10277 | 10666 | 1344 |
| 17 | IOL.tw. | 5267 | 6179 | 998 |
| 18 | or/14-17 | 16543 | 16814 | 1954 |
| 19 | Astigmatism/ | 4870 | 6290 | 296 |
| 20 | astigmat$.mp. | 7730 | 7656 | 831 |
| 21 | 19 or 20 | 7730 | 7656 | 831 |
| 22 | 8 and 12 and 18 | 7998 | 8939 | 1298 |
| 23 | 19 and 22 | 34 | 78 | 4 |
| 24 | 21 and 23 | 652 | 722 | 129 |
